# Vaccination with a live-attenuated small-colony variant improves the humoral and cell-mediated responses against *Staphylococcus aureus*

**DOI:** 10.1371/journal.pone.0227109

**Published:** 2019-12-27

**Authors:** Julie Côté-Gravel, Eric Brouillette, François Malouin

**Affiliations:** Centre d’étude et de Valorisation de la Diversité Microbienne (CEVDM), Département de Biologie, Faculté des Sciences, Université de Sherbrooke, Sherbrooke, Canada; University of South Dakota, UNITED STATES

## Abstract

*Staphylococcus aureus* is known to produce persistent and chronic infections in both humans and animals. It is recognized that small-colony variants (SCVs), which produce higher levels of biofilm and that are capable of intracellular persistence, contribute to the chronicity or recurrence of infections and that this phenotype is inherent to the pathogenesis process. Prevention of *S*. *aureus* infections through vaccination has not yet met with considerable success. Some of the current vaccine formulations for *S*. *aureus* bovine mastitis consist of inactivated *S*. *aureus* bacteria, sometimes combined to *E*. *coli* J5. As such, the stimulation of cell-mediated immunity by these vaccines might not be optimal. With this in mind, we recently engineered a genetically stable double mutant SCV (*ΔvraGΔhemB*), which was highly attenuated in a mastitis model of infection. The present work describes the immune responses elicited in mice by various experimental vaccine compositions including the live-attenuated SCV double mutant and its inactivated form, combined or not with inactivated *E*. *coli* J5. The live-attenuated SCV was found to provoke a strong and balanced humoral response in immunized mice, as well as strong proliferation of *ex-vivo* stimulated splenocytes isolated from these animals. These splenocytes were also found to release high concentration of IL-17 and IFN-γ when compared to every other vaccination formulation. Inversely, the inactivated whole-cell vaccine, alone or in combination with the *E*. *coli* J5 bacterin, elicited lower antibody titers and failed to induce Th1 or Th17 cell-mediated responses in the splenocyte proliferation assay. Our results suggest that live-attenuated SCVs can trigger host immunity differently than inactivated bacteria and could represent a suitable vector for inducing strong humoral and cell-mediated immune responses, which are crucial for protection. This could represent an important improvement over existing vaccine formulations for preventing *S*. *aureus* bovine mastitis and other infections caused by this pathogen.

## Introduction

*Staphylococcus aureus* is an opportunistic pathogen that has the ability to affect several tissues and organs in human and animal hosts, and to induce both acute and chronic types of infections. This pathogen possesses an abundance of virulence factors, with many of them contributing to its ability to persist in host cells and tissues, resist or counter drug therapies and evade host immune responses [1]. The development of new alternatives to fight this pathogen is becoming increasingly urgent. Vaccine development against *S*. *aureus* for either humans or animals has been unsuccessful to date. Challenges include the diversity of strains that can cause infections, the ability of *S*. *aureus* to counteract host immune defenses [2] and insufficient understanding of the type of immune defense required for efficient protection against such a polyvalent pathogen with both extracellular and intracellular lifestyles [3].

*Staphylococcus aureus* is the most commonly found pathogen in clinical bovine mastitis [4], but it is also the cause of subclinical, persistent and difficult-to-treat intramammary infections (IMIs) [5,6]. Bovine mastitis affects animal health, milk production and quality, and challenges the economic efficiency of dairy producers [7]. Spreading of undetected subclinical IMIs during milking maintains a reservoir in the herd and is a difficult problem that may be better tackled through preventive interventions. Vaccines could represent the ideal prevention tools to reduce the incidence of new cases of IMIs and improve milk production and quality.

Vaccine development for *S*. *aureus* mastitis is challenging [8]. Commercially available vaccines for the prevention of *S*. *aureus* mastitis consist of inactivated bacteria or bacterin-based products, including a lysed whole cell vaccine of capsular *S*. *aureus* serotypes (Lysigin, Boehringer Ingelheim Vetmedica, Inc.)[9] and a multivalent inactivated vaccine (StartVac® or TopVac®, Hipra, Spain) composed of *E*. *coli* J5 and a *S*. *aureus* strain that expresses slime-associated antigens part of the biofilm extracellular matrix [10]. Although the use of whole bacterins provides a selection of antigens that are suitable for raising an immune response, the success of such an approach is highly dependent on the diversity and type of *S*. *aureus* strains present in herds. Additionally, it is still unclear if such multivalent inactivated vaccines have the ability to raise the adequate type of immune response to protect against *S*. *aureus* infections, as they have been shown to generate mostly humoral responses against this pathogen [11]. Antibody-based immunity may be important but is likely insufficient for protection against *S*. *aureus* chronic infections without the contribution of a cell-mediated response [12,13].

In human and veterinary medicine, *S*. *aureus* small-colony variants (SCVs) contribute to therapeutic failures and are frequently isolated from chronic infections [14,15]. SCVs are adapted for long-term persistence and are capable of high biofilm production [16,17] and invasion of host cells [18,19], shielding the bacteria from drugs and the host immune system. Several SCV isolates from dairy cattle with a history of chronic mastitis have been previously reported [20,21] but are usually overlooked in routine milk culture procedures because of their slow growth and atypical colony appearance. Recurrent antibiotic treatments and internalization of *S*. *aureus* in mammary gland epithelial cells may indeed represent favorable conditions to the generation of SCVs [20], and potentially explain some of the relatively low cure rates observed for *S*. *aureus* IMIs [22]. Hence, SCVs can add important contributions to the persistence in infections; however, their natural slow-growing phenotype and low expression of dissemination virulence factors could also be exploited in vaccines development, following further attenuation.

Genetically stable *S*. *aureus* SCVs can be engineered through the deletion of gene *hemB* [23] to prevent reversion to the virulent prototypic phenotype that expresses numerous exotoxins. In a previous work [24], we have constructed a double mutant by the complete deletion of *hemB* in addition to the inactivation of gene *vraG* (SACOL0720), which was shown to be important for full virulence during bovine IMIs [25]. The Δ*vraG*Δ*hemB* SCV strain was shown to be greatly attenuated in a bovine epithelial mammary cells invasion/persistence assay and in the murine intramammary infection (IMI) model [24]. Additionally, high doses of subcutaneous injections could be achieved in mice without provoking any sign of local inflammation or adverse effect. Such a strain could therefore be used as a live-attenuated vaccine. Immunization of mice using increasing concentrations of Δ*vra*GΔ*hemB* yielded a substantial rise of specific antibody titers against a variety *S*. *aureus* strains isolated from bovine mastitis, including strains from the major *spa* types found in Canada and elsewhere in the world [24]. Live-attenuated vaccine that mimic natural infections are known to stimulate the immune system in a powerful way, producing high affinity serum and mucosal antibodies as well as different effectors of cell-mediated immunity due to the recognition of microbial viability by the innate immune system [26].

In the present work, we describe the characterization of the humoral and cellular responses that develop following vaccination with the live-attenuated Δ*vra*GΔ*hemB* SCV vaccine. These responses were compared to that achieved with its inactivated version, alone or in combination with inactivated *E*. *coli* J5 bacteria. *E*. *coli* J5 is a well-known O polysaccharide mutant, which exposes its lipopolysaccharide (LPS) core region and that has been used for producing cross-reacting antibodies against *Enterobacteriaceae* and Gram-negative bacteria [27]. This characterization could help to attain a better understanding of the factors behind the suboptimal protection currently achieved with vaccines that use inactivated *S*. *aureus* or that combine *S*. *aureus* antigens to Gram-negative bacterins. Results revealed the potential advantages of developing alternate strategies such as immunization with live-attenuated *S*. *aureus* strains, particularly in order to improve cell-mediated immunity and protection against *S*. *aureus*.

## Materials and methods

### Ethics statement

The animal experiments were conducted following the guidelines of the Canadian Council on Animal Care and the institutional ethics committee on animal experimentation of the Faculté des Sciences of Université de Sherbrooke. The institutional ethics committee on animal experimentation of the Faculté des Sciences of Université de Sherbrooke approved this study.

### Bacterial strains and *S*. *aureus* live-attenuated vaccine

Unless otherwise stated, *S*. *aureus* and *Escherichia coli* J5 strains were grown in tryptic soy broth (TSB) and agar (TSA) (BD, Mississauga, ON, Canada). The *E*. *coli* J5 strain was obtained from the American Type Culture Collection (ATCC 43745). The development of the *S*. *aureus* double mutant strain Δ*vraG*Δ*hemB* was described elsewhere [24]. For the preparation of bacterial vaccine samples, *S*. *aureus* Δ*vraG*Δ*hemB* colonies previously grown on brain heart infusion agar (BHIA) (BD) were washed twice in ice cold PBS (Wisent, St-Bruno, QC, Canada) and suspended in PBS containing 15% glycerol, then were aliquoted and kept at -80°C until subsequent use. The concentration of *S*. *aureus* Δ*vraG*Δ*hemB* was assessed by serial dilutions in PBS and plating on TSA, and suspensions were freshly adjusted to 5 × 10^7^ CFU/ml of PBS on the immunization day.

### Inactivation of bacteria

Bacterial suspensions of *S*. *aureus* Δ*vraG*Δ*hemB* and *E*. *coli* J5 were also heat-killed to obtain an inactivated version of the vaccines for immunization and for stimulation of mice splenocytes in subsequent assays. Different heat inactivation treatments were evaluated to select the lowest temperature and time exposure to attain total killing of bacteria. *S*. *aureus* Δ*vraG*Δ*hemB* and *E*. *coli* J5 previously grown and prepared in suspensions of 5 × 10^7^ CFU/ml in PBS were treated for 10, 20 or 30 min at 65°C and 5 min at 80°C. Undiluted bacterial suspensions were then plated (200 μl) in triplicate on BHIA and incubated for 48 h at 37°C to confirm inactivation (no growth). For *E*. *coli* J5, 10 min at 65°C was found to be sufficient for complete inactivation of bacteria whereas 20 min at the same temperature was necessary for killing of *S*. *aureus* Δ*vraG*Δ*hemB*. Inactivated bacteria were stored at– 80°C until subsequent use.

### Preparation of *S*. *aureus* cell extract

Preparation of a *S*. *aureus* Δ*vraG*Δ*hemB* whole cell extract was done as previously described with some modifications [28]. Briefly, overnight bacterial cultures were diluted 1/1000 in fresh BHI broth, and then incubated at 35°C (225 rpm) until an *A*_600nm_ of ~ 0.8 was reached. Bacterial cells were centrifuged, and pellets were washed twice in ice-cold PBS and resuspended in a ratio of 5 ml of PBS per ml of pellet. Bacterial suspensions were then treated with 100 μg of lysostaphin (Sigma-Aldrich, Oakville, ON, Canada) per ml of pellet for 1 h at 37°C, and then 3 μg of protease inhibitor cocktail (Sigma-Aldrich), 8 μg of RNAse A (Sigma-Aldrich) and 8 μg of DNAse (Qiagen, Toronto, ON, Canada) per ml of pellet were added to the suspension. After 30 min at room temperature, cells were mechanically disrupted by 3 to 4 passages in a SLM Aminco French Pressure cell disrupter, and then centrifuged at 12,000 × *g* at 4°C for 10 min to remove unbroken cells. The supernatant was collected and used as the whole cell extract. Total protein concentration was determined by the bicinchoninic acid method (BCA) Protein Assay Kit (Thermo Fisher Scientific, Ottawa, Canada).

### Immunization of mice

**Fig 1:**
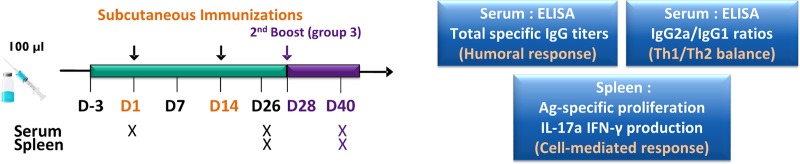
Experimental design and timeline of immunizations and sample collection. CD-1 female mice were immunized by subcutaneous injections (100 μl, arrows) two weeks apart at day 1 (D1) and day 14 (D14) as well as at day 28 (D28), for mouse group 3 only. CD-1 mice were divided into 6 groups (*n* = 5 mice per group), see the Materials and Methods section. Group 1 (SCV Inac) received the heat-inactivated *S*. *aureus* Δ*vraG*Δ*hemB*; group 2 (SCV Live), received the live-attenuated *S*. *aureus* Δ*vraG*Δ*hemB*; group 3 (SCV Live 3), received the same regimen as group 2, but with an additional boost immunization 2 weeks after the 2^nd^ immunization as illustrated in purple on this timeline; group 4 (SCV Inac + J5), received a combination of the heat-inactivated *S*. *aureus* Δ*vraG*Δ*hemB* and the heat-inactivated *E*. *coli* J5; group 5 (SCV Live + J5), received a combination of the live-attenuated *S*. *aureus* Δ*vraG*Δ*hemB* and the heat-inactivated *E*. *coli* J5; and group 6 received only PBS (non-vaccinated control group). The boxes identify the tests performed for serum and spleen samples taken at the indicated time points (X).

CD-1 female mice weighing 16–18 g were obtained on demand from Charles River Laboratories Inc. (Saint-Constant, QC, Canada). After arrival at our animal facilities, 5 mice per filtered cage were randomly assigned, and had *ad libitum* access to food and water. Prior to each experimental injection or blood samplings, animals were anesthetized by intramuscular injection of a mixture of ketamine and xylazine at 87 and 13 mg per kg of body weight. Throughout the experimentation, animal health was daily monitored by a certified animal care technician. Mice were immunized by two subcutaneous injections (100 μl) two weeks apart, following the timeline illustrated in Fig 1. CD-1 mice were divided into 6 groups (*n* = 5 mice per group): group 1 (SCV Inac) received the heat-inactivated *S*. *aureus* Δ*vraG*Δ*hemB* (5 × 10^7^ CFU that were heat-killed); group 2 (SCV Live), received the live-attenuated *S*. *aureus* Δ*vraG*Δ*hemB* (5 × 10^7^ CFU); group 3 (SCV Live 3), received the same regimen as group 2, but with an additional boost immunization (3 injections in total) 2 weeks after the 2^nd^ immunization as illustrated in Fig 1; group 4 (SCV Inac + J5), received a combination of the heat-inactivated *S*. *aureus* Δ*vraG*Δ*hemB* and the heat-inactivated *E*. *coli* J5 (5 × 10^7^ CFU of each heat-killed bacterial suspensions); group 5 (SCV Live + J5), received a combination of the live-attenuated *S*. *aureus* Δ*vraG*Δ*hemB* (5 × 10^7^ CFU) and the heat-inactivated *E*. *coli* J5 (5 × 10^7^ CFU); and group 6 (PBS), received 100 μl PBS. Blood samples were collected prior to the first injection and 10 days after the final boost. The blood samples were allowed to clot at room temperature for an hour and were then centrifuged at 2,000 × *g* for 10 min at 4°C. The sera were harvested and kept at -20°C until subsequent analysis. Ten days after the final boost, mice were euthanized by cervical dislocation after deep anesthesia with the ketamine and xylazine mixture (see above), and spleens were aseptically harvested to isolate fresh splenocytes intended for antigen-specific cell proliferation and cytokine production assays.

### ELISAs

Serum total IgG and IgG1/IgG2a isotypes were detected by ELISA against the *S*. *aureus* Δ*vraG*Δ*hemB* whole cell extract to compare the systemic humoral response generated by the different vaccine versions as previously described [24]. Briefly, Nunc MaxiSorpTM 96-well plates (Thermo Fisher Scientific) were coated with 100 μl of whole *S*. *aureus* cell extract (10 μg/ml diluted in carbonate/bicarbonate buffer, Sigma) and incubated overnight at room temperature. The plates were then saturated with PBS containing 5% skim milk for 1 h at 37°C, followed by a second blocking step with the addition of 5% porcine serum to prevent unspecific interactions with *S*. *aureus* protein A and other staphylococcal immunoglobulin binding proteins [24]. One hundred microliters of four-fold serial dilutions of the sera in dilution buffer (PBS with 2% milk, 2% porcine serum and 0.025% Tween 20 [Sigma]) were loaded onto the plates and incubated for 1 h at 37°C. Plates were then washed three times with PBS containing 0.05% Tween 20, and loaded with 100 μl of horseradish peroxidase (HRP)-conjugated goat anti-mouse IgG, IgG1 or IgG2a (Jackson ImmunoResearch Laboratories Inc., West Grove, PA) previously diluted 1:5000 in the dilution buffer. After 1 h of incubation at 37°C followed by 3 washes, peroxidase activity was detected with the 3,3′,5,5′-tetramethylbenzidine (TMB) reagent at 450 nm using an Epoch microplate reader (Biotek Instruments Inc.) after the addition of 1M H_2_SO_4_ (KPL Inc., Gaithersburg, MD) according to the manufacturer’s recommendations.

### Isolation of murine splenocytes

After animals were sacrificed under anesthesia, spleens were harvested aseptically and kept in ice cold Dulbecco's phosphate-buffered saline (DPBS; Wisent). The excised spleens were cut into small pieces and were pressed through 100-μm nylon cell strainers using the plunger end of a syringe. Cells were then washed with DPBS and centrifuged at 1,800 rpm for 5 min. Cell pellets were suspended in 1 ml of pre-warmed red blood cell lysis solution (Sigma-Aldrich) and incubated for 2 min at 37°C. After the lysis was stopped by the addition of 30 ml DPBS, cells were centrifuged and suspended in fresh DPBS, and cell count and viability were verified using trypan blue exclusion. Splenocytes were then immediately used for the proliferation and cytokine production assays.

### Splenocyte proliferation assay

Freshly isolated splenocytes were used for a proliferation assay in order to measure the specific cellular response of immunized mice after stimulation with inactivated bacteria. Briefly, cells were suspended in complete Roswell Park Memorial Institute (RPMI) medium supplemented with 10% fetal bovine serum, glutamine, antimycotic-antibiotic solution (1 X of the Penicillin, Streptomycin and Amphotericin B solution from Wisent), non-essential amino acids solution and 2-mercaptoethanol (Sigma-Aldrich). Cell culture reagents were all purchased from Wisent. Cells were adjusted to a concentration of 5 × 10^5^ cells/ml and were distributed in 96-wells culture microplates. They were then stimulated with 5 × 10^5^ CFUs of heat-inactivated *S*. *aureus* Δ*vraG*Δ*hemB* or *E*. *coli* J5, 5 μg/ml of Concavalin A (Sigma-Aldrich) or cell culture medium (untreated control). Splenocytes were incubated and proliferation was allowed for 60 h at 37°C in a humidified incubator with 5% CO_2_. Cells were then centrifuged, and supernatants were aliquoted and kept at -20°C for cytokine production analysis. Cell pellets were immediately used for the metabolic activity assay, which measures the reduction of 3-[4,5-dimethylthiazol-2-yl]-2,5 diphenyl tetrazolium bromide (MTT) into an insoluble formazan product in metabolically active cells. Briefly, cells were suspended in warm DPBS and 10 μl MTT solution (5 mg/ml) was added before an incubation period of 2 h at 37°C. Cells were then centrifuged, and an acidic solvent solution of 16% SDS and 40% Dimethylformamide, pH 4.7, was added to lyse the cells and solubilize the formazan crystals. The *A*_570nm_ of the samples were then measured with a correction at *A*_650 nm_ using an Epoch microplate reader (Biotek Instruments Inc.). All assays were performed in triplicate. Proliferation was then expressed as the ratio of absorbance of treated cells on untreated cells for each mouse-specific splenocytes.

### Cytokine production assay

Release of IL-17a and IFN-γ into the supernatant of splenocyte cultures was quantified by a capture enzyme linked immunosorbent assay (ELISA) using DuoSet sandwich ELISA kits (R&D systems, Minneapolis, Mn) and following the manufacturer’s recommendations. Peroxidase activity was detected by adding the 3,3′,5,5′-tetramethylbenzidine (TMB) reagent (KPL Inc., Gaithersburg, MD) following the usual procedure of ELISAs.

### Statistical analysis

Statistical analysis was carried out using the GraphPad Prism software (v.6.02). Total IgGs, IgG2a and IgG1 titers as well as splenocyte proliferation ratios were transformed in base 10 logarithm values before being used for statistical analysis. Specific statistical tests used for the analysis of each experiment and statistical significance are specified in the legend of each figure.

## Results

### Immunization of mice with live-attenuated SCV stimulates a strong and specific humoral response against *S*. *aureus* compared to inactivated bacteria

As the live-attenuated Δ*vraG*Δ*hemB* SCV vaccine has the capacity to elicit strong and specific humoral responses against a variety of mastitis associated *S*. *aureus* strains without the use of any adjuvants [24], we hypothesized that this high immunogenicity is linked to the live nature of the vaccine. To investigate the impact of this live vaccine composition on both humoral and cell-mediated responses in mice, we compared a group of mice immunized by two injections of 5x10^7^ CFU the live-attenuated Δ*vraG*Δ*hemB* SCV strain to: (i) a group receiving three injections, (ii) a group immunized with inactivated Δ*vraG*Δ*hemB* SCV bacteria, and (iii) mice receiving a combination of inactivated *E*. *coli* J5 strain with the live or inactivated SCV (Fig 1).

**Fig 2:**
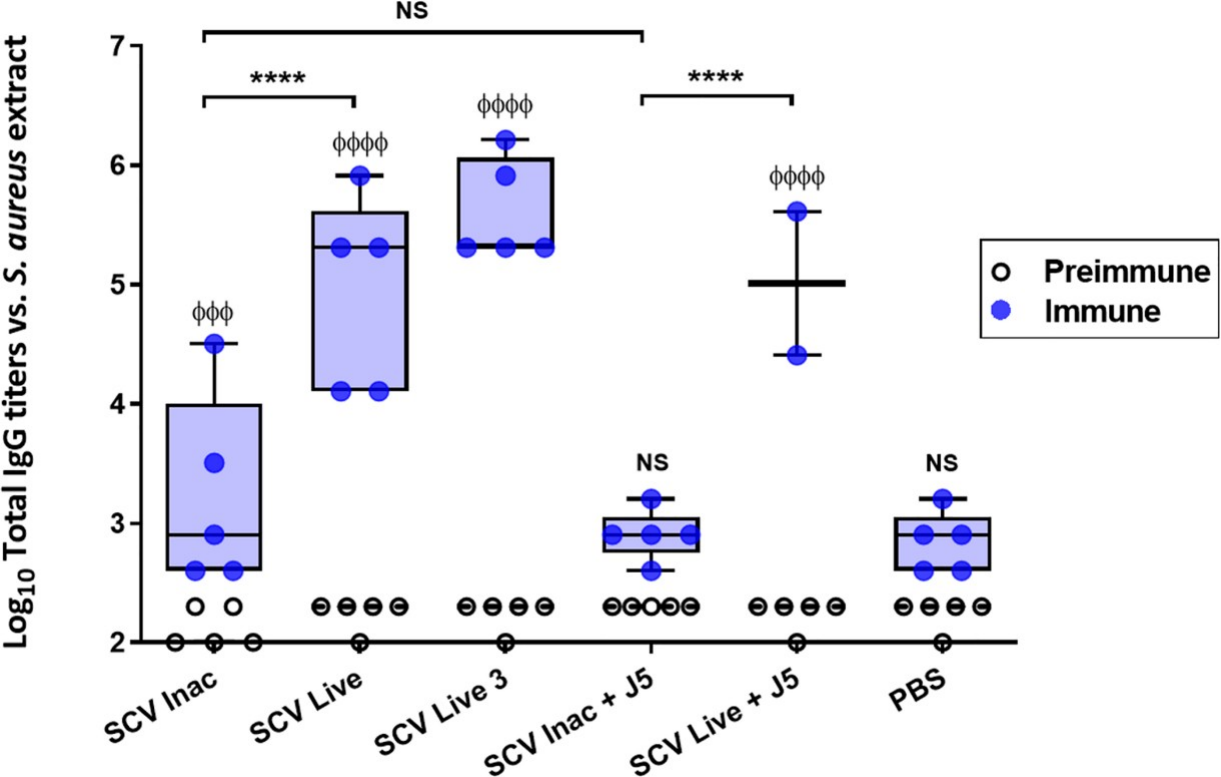
Immunization of mice with the live-attenuated double mutant SCV (Δ*vraG*Δ*hemB*) stimulates a strong humoral response against *S*. *aureus*. The six groups of mice (n = 5 mice per group) are defined in the Materials and Methods section and the immunization schedule is shown in Fig 1. Sera were collected before priming immunization (preimmune) and 10 days after the boost immunization (immune). Each dot represents the total IgG titer of one mouse against *S*. *aureus* Δ*vraG*Δ*hemB* whole cell extract, and boxes (upper and lower boundaries) and vertical lines (whiskers) indicate interquartile distances and ranges, respectively. Medians are represented by thick horizontal lines for immune titers and dashed lines for preimmune titers. Immune titers were compared to their corresponding preimmune titers (Two-way ANOVA and Sidak's multiple comparisons test: φφφφ P ≤ 0.0001; φφφ P ≤ 0.001; NS, not significant), as indicated on the top of the corresponding box, or to other groups (Two-way ANOVA and Tukey's multiple comparisons test: ****P ≤ 0.0001; NS, not significant).

Serum total IgGs of immunized mice were assayed in ELISAs for binding to whole cell extracts of *S*. *aureus*. For every group of mice but one (group 5; live attenuated *S*. *aureus* combined to inactivated *E*. *coli* J5), the vaccine subcutaneous injections triggered no adverse effects in mice such as modification of behavior, signs of inflammation or necrosis at the immunization site throughout the immunization period. However, the addition of inactivated J5 bacteria to live SCV in group 5 led to the development of important signs of inflammation in the upper back area near the site of injection in 3 out of 5 mice, 3 to 4 days after the priming injection. The inflammation was sustained for the next few days and was considered too important for the welfare of the animals; thus those 3 mice were euthanized. The two remaining mice developed a moderate inflammation that subsided before the second immunization. The boost immunization did not lead to higher inflammation. Fig 2 illustrates the *S*. *aureus* antigen-specific total IgG titers that were measured in preimmune and immune serum samples of immunized mice. For each vaccination group in which the live vaccine was used (groups 2, 3 and 5), significantly higher IgG titers were detected in the immune samples as compared to their corresponding preimmune sera (P ≤ 0.0001). Heat-inactivated SCV also led to higher IgG titers (P = 0.0006), but these higher immune titers were not observed when inactivated SCV bacteria were combined with inactivated J5 (group 4). Indeed, there was no difference between the preimmune and immune sera for the groups vaccinated with the combination of inactivated SCV and J5 or the PBS control. Conversely, groups that received the live SCV version of the vaccine were all shown to develop statistically higher humoral responses than inactivated formulations or PBS control immunization (P ≤ 0.0001), independently of the combination with inactivated *E*. *coli* J5. These results demonstrate that the live vaccine is quite efficient on its own in its ability to raise high antibody titers. These titers are also higher than that obtained with heat-inactivated bacteria. For mice immunized using an additional boost of the live SCV vaccine, sera yielded a consequential rise of IgG titers (Fig 2) against *S*. *aureus* antigens, but this trend was not found to be statistically different to that obtained with the two-dose immunization (P = 0.1270). This suggests that the live strain is already highly immunogenic after two injections.

Taken together, these results clearly show that (i) immunization with the Δ*vra*GΔ*hemB* live vaccine can raise a higher humoral response against *S*. *aureus* antigens than its inactivated counterpart, and that (ii) an additional boost immunization (group 3, three injections in total) yields higher titers that are not significantly different than with two injections (group 2). Furthermore, this humoral response against *S*. *aureus* (iii) is not enhanced by the combination with *E*. *coli*. On the contrary, addition of the inactivated J5 to the inactivated SCV vaccine yielded lower titers of *S*. *aureus* specific IgGs compared to that obtained using the inactivated SCV alone. Also, when combined to the live SCV vaccine, addition of the inactivated J5 resulted in acute inflammatory responses in mice.

### Immunizations with the live-attenuated SCV improves the Th1/Th2 immune response balance against *S*. *aureus*

**Fig 3:**
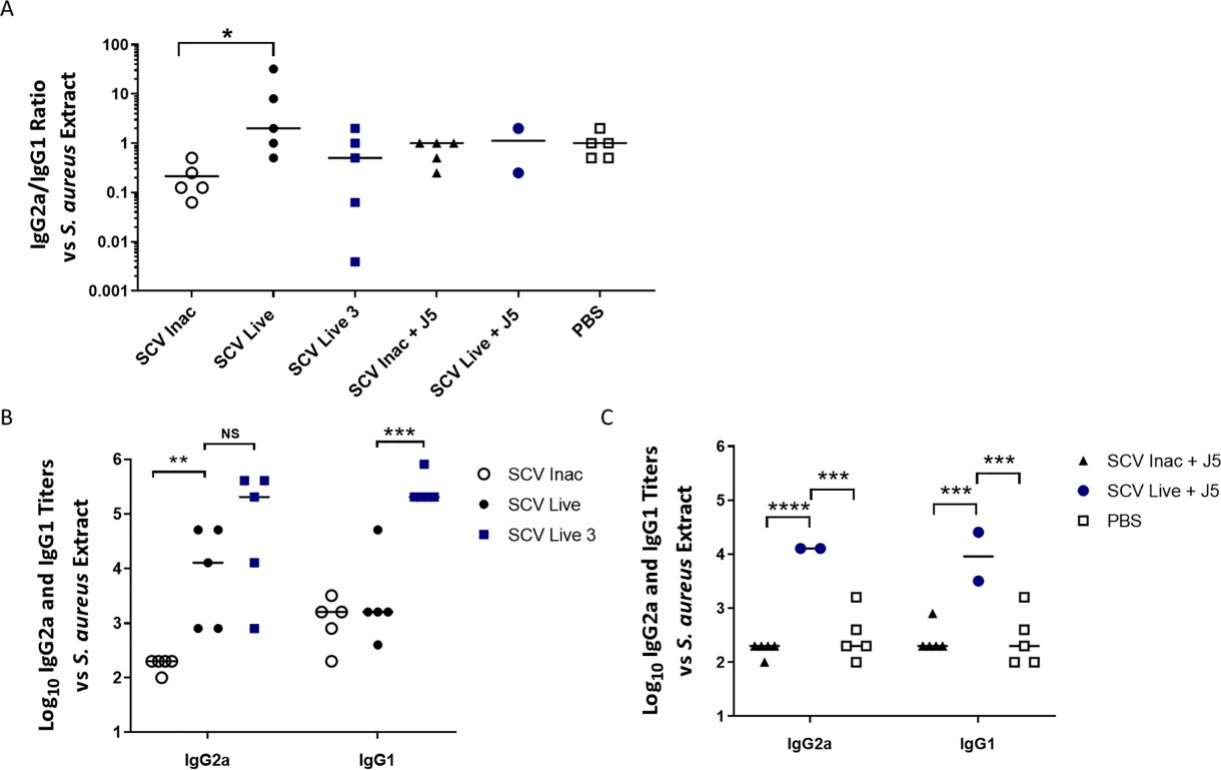
Th1/Th2 immune response balance of mice immunized with the live-attenuated double mutant SCV (Δ*vraG*Δ*hemB*) or inactivated bacteria. The six groups of mice shown in (A), (B) and (C) are defined in the Materials and Methods section and the immunization schedule is shown in Fig 1. Specific IgG2a/IgG1 ratios (A) and IgG2a and IgG1 titers (B-C) of mice against Δ*vraG*Δ*hemB* whole cell extracts. Each dot represents the IgG2a/Ig1 ratio or the immune IgG2a and IgG1 titer of one mouse. Ratios were calculated using the specific IgG2a/IgG1 titers of each mouse. Medians are represented by horizontal lines. Ratios or titers were compared between each group (A: Kruskal-Wallis test with Dunn’s multiple comparison test; *P ≤ 0.05; B-C: Two-way ANOVA and Sidak's multiple comparisons test; ****P ≤ 0.0001; ***P ≤ 0.001; **P ≤ 0.01; NS, not significant).

In an effort to further characterize the immune response elicited by the various vaccines, the *S*. *aureus* specific IgG2a and IgG1 isotypes titers were measured as markers for the resulting balance between the Th1 and Th2 responses [29]. Since *S*. *aureus* has the capacity to invade and survive in non-phagocytic host cells [19] and that antibodies alone are insufficient to protect against this pathogen [3,30], we sought to find out what vaccine formulation could induce a balanced Th1/Th2 type response, hence a higher IgG2a/IgG1 ratio. Fig 3A shows that the IgG2a/IgG1 ratio is significantly higher in immune sera from the live-attenuated SCV vaccine group than that obtained by using the inactivated SCV vaccine, suggesting enhanced activation of the cell-mediated immunity pathway in these mice. A lower ratio was also obtained with all the other groups, indicating an excess in IgG1 or equivalent quantities of the two isotypes in the sera of these mice; however, this trend was not found to be statistically significant. Besides, in inactivated SCV + J5 and PBS groups, very low immune IgG titers are likely the cause of this limited differentiation between one or the other isotype (Fig 3C). In the same way, addition of the inactivated J5 bacterin to the live-attenuated SCV vaccine (group 5) had no significant effect on the IgG2a/IgG1 ratio when compared with the live vaccine alone. All the mice that received live SCVs produced significantly higher IgG2a titers (Fig 3B and 3C). Besides, the apparent reduction in the IgG2a/IgG1 ratio for the live attenuated vaccine that was provided by three injections (Fig 3A, Live 3), was mainly due to the very high production of IgG1 subsequent to the last boost immunization, and not to lower IgG2a titers as shown in Fig 3B.

### Live-attenuated vaccine induces the proliferation of *S*. *aureus*-specific Th1 and Th17 cell-mediated immunity actors

**Fig 4:**
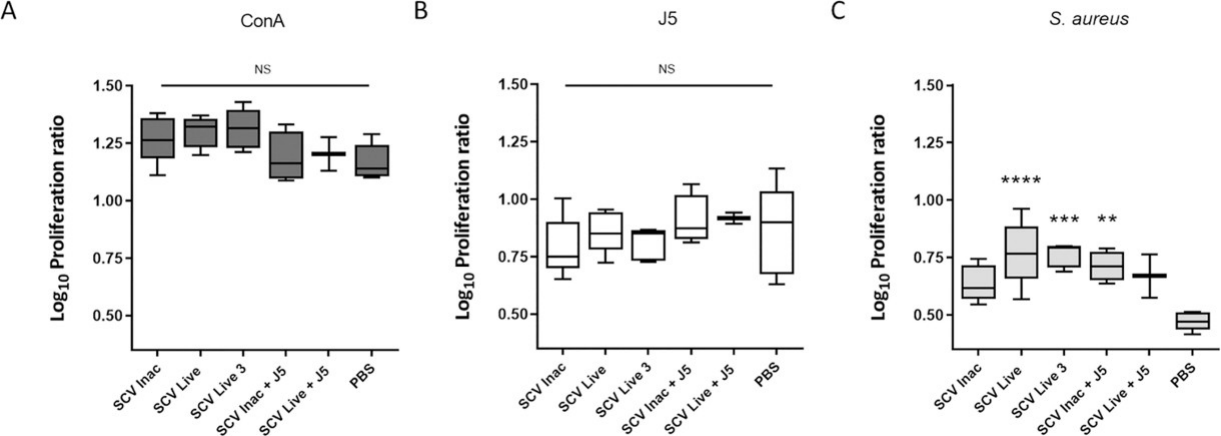
*S*. *aureus*-specific proliferation of splenocytes from vaccinated mice. Cells were stimulated for 60 h with Concanavalin A (A), heat-inactivated *E*. *coli* J5 bacteria (B) or heat-inactivated *S*. *aureus* SCV Δ*vraG*Δ*hemB* (C). Each box represents the interquartile distances and ranges of combined results of Log_10_ proliferative ratio of cells isolated from spleens of mice from one group, normalized with unstimulated cells. Horizontal lines represent the medians. Each group of immunized mice were compared to the PBS group (Two-way ANOVA and Tukey's multiple comparisons test: ****P ≤ 0.0001; ***P ≤ 0.001; **P ≤ 0.01; NS, not significant). The six groups of mice shown in (A), (B), and (C) are defined in the Materials and Methods section and the immunization schedule is shown in Fig 1.

In order to evaluate and compare the cell-mediated response elicited by the different vaccine formulations, we collected spleens from sacrificed mice 10 days after the final boost injection. Splenocytes isolated and cultured from vaccinated mice were cultured and assayed for proliferation upon exposure to the inactivated *S*. *aureus* Δ*vraG*Δ*hemB* and *E*. *coli* J5. The spleen cells comprise various immunity actors, mainly B and T lymphocytes, but also macrophages, dendritic cells, etc. Proliferation was determined by the ratio of metabolically active cells from every stimulated and unstimulated spleen for each individual mouse. These stimulations were done in triplicate for every mouse and Fig 4 presents the combined results for all of the mice in one group. Statistical differences arising from these proliferation ratios were calculated by comparing each group of mice to the PBS control group. Mitogenic positive control concanavalin A provoked high proliferation of stimulated splenocytes in every group of mice, as expected (Fig 4A). Unfortunately, stimulation of splenocytes with inactivated *E*. *coli* J5 led to unspecific proliferation (Fig 4B), as seen by the PBS control group being fairly high, indicating possible interactions of the LPS or other molecules from the J5 strain with cell activation. Cells that were stimulated with inactivated *S*. *aureus* Δ*vra*GΔ*hemB*, however, showed specific proliferation that was well distinguished between the vaccination groups. The Δ*vra*GΔ*hemB* live vaccines (either 2 or 3 doses), led to significantly higher cell proliferation ratios compared to that obtained with the PBS-immunized control (Fig 4C).

**Fig 5:**
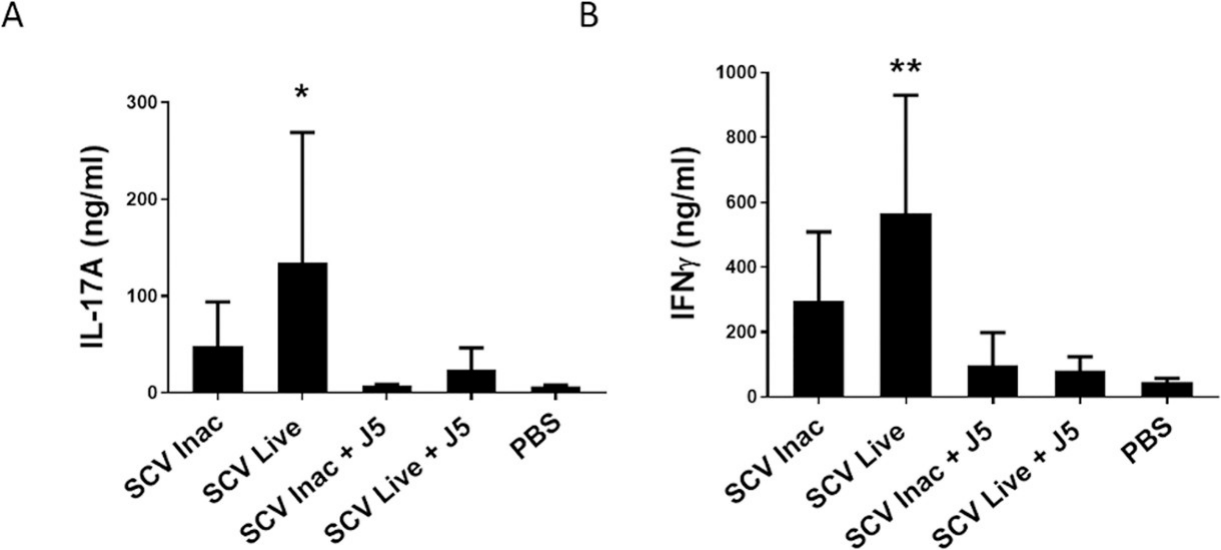
*S*. *aureus*-specific IL-17A and IFN-γ responses elicited by splenocyte proliferation of immunized mice. The six groups of mice shown in (A) and (B) are defined in the Materials and Methods section and the immunization schedule is shown in Fig 1. Immunized mice were sacrificed 10 days after the second (boost) immunization, and splenocytes were prepared and stimulated with the heat-inactivated *S*. *aureus* Δ*vraG*Δ*hemB* SCV for 60 h in RPMI medium. Cells were centrifuged and supernatants were collected. IL-17A (A) and IFN-γ (B) concentrations from cell culture medium quantified by sandwich ELISA and compared to standard curves of recombinant cytokines. Data are expressed as means ± standard errors and statistical differences with the PBS-immunized control mice are shown (Kruskal–Wallis ANOVA with Dunn’s multiple comparison test: **P ≤ 0.005; *P ≤ 0.05).

The inactivated *S*. *aureus* strain combined with the J5 bacterin also generated significant spleen cells proliferation, but to a lower extent. The splenocytes of mice from that group were not found to produce significant amount of Il-17a or IFN-γ in the cell culture media following stimulation (Fig 5). In fact, only the immunization with the *S*. *aureus* Δ*vra*GΔ*hemB* live vaccine led to a significant production of IL-17A and IFN-γ by spleen lymphocytes stimulated by heat-inactivated *S*. *aureus* (Fig 5). Interestingly, vaccination with the inactivated J5 bacterin combined to the live-attenuated *S*. *aureus* vaccine slightly suppressed the spleen cells proliferation obtained by using the live vaccine alone (Fig 4A), despite their equally robust humoral response (Fig 2). Likewise, cytokine production of splenocytes from this group was also found to be very low compared to the live vaccine.

Overall, these results of proliferation and production of Th17- and Th1-associated cytokines from *S*. *aureus*-stimulated splenocytes obtained from mice vaccinated with the live-attenuated vaccine provide further evidence of the balanced humoral and cellular immunity responses triggered by the live vaccine.

## Discussion

Vaccine development efforts against *S*. *aureus* suggest that vaccine-induced antibodies may be important but frequently appear insufficient for achieving protection against this pathogen [31]. Currently, as perceived from studies in humans and mice [30,32,33], it is assumed that good Th1 and Th17 responses combined to humoral immunity may be required to obtain at least some efficacy against *S*. *aureus*, although no specific marker for protection was found thus far [3]. The results of this study support previous observations that live vaccines are often better than their inactivated counterparts at inducing strong and balanced immune responses which could contribute to long-term protection [34]. In order to evaluate the influences of bacterial viability and the combination with a *E*. *coli* bacterin on the development of a strong and balanced *S*. *aureus*-specific immune response, we compared the humoral and cell-mediated immunity generated by immunization with several *S*. *aureus* SCV-based vaccine formulations in mice.

The benefits of the live-attenuated SCV strain were apparent when compared to the same dose of heat-killed bacteria: the live-attenuated vaccine induced higher IgG titers and significantly improved the IgG2a/IgG1 antibody ratio against *S*. *aureus* in contrast to that observed with the heat-inactivated vaccine. In mice, IgG2a/IgG1 titers ratios are good indicators of the relative importance of the Th1 and Th2 pathways, since these isotypes are produced under the influence of different cytokines during the rise of the acquired immune response. The balanced immune response triggered in mice that received the live vaccine also provoked a strong proliferation of *ex-vivo* stimulated splenocytes isolated from these animals. These splenocytes were also found to release higher concentration of IL-17 and IFN-γ when compared to every other immunization groups. Despite the fact that no adjuvant was added to the different formulations used and compared in this study, the live SCV vaccine was highly immunogenic by itself, as was formerly observed [24]. This effect was in contrast to the heat-killed formulation, so it is likely that the promotion of higher IgG titers and Th1 and Th17-oriented responses resulted from underlying mechanisms specific to viable organisms.

Vaccines to prevent *S*. *aureus* infections, in the case of bovine mastitis, have either shown insufficient protection to be accepted for commercialisation or currently only offer limited benefits to be widely used. Whole inactivated bacteria (bacterins) do have the potential to provide antigens that are suitable for raising an immune response, but their protective success is highly dependent on the compatibility and virulence of strains that are present in herds. Moreover, it is apparent that these formulations give rise to immune responses mostly composed of antibody components that can only partly diminish the virulence and/or clinical symptoms of *S*. *aureus* infections but are not sufficient on their own to prevent colonization [11]. As such, the commercially available StartVac vaccine prepared from killed *S*. *aureus* and *E*. *coli* J5 bacterins, aiming at controlling bovine mastitis, has met with contrasting conclusions in recent field trials studies taking a look at its efficacy against *S*. *aureus* IMIs. Although StartVac had moderate success in reducing the incidence of new *S*. *aureus* infections [35] or the severity of clinical infections [36], it was also shown to lack protection efficacy against new *S*. *aureus* IMIs and had no beneficial effect on milk production or survival rates of vaccinated cows in other herds [37]. Differences in herd structure, management and production level, together with differences in *S*. *aureus* strains type between countries and regions were presented as the probable causing factors for these discrepancies. Here we saw that the combination with J5 bacterin had no beneficial effect or diluted the strength and specificity of humoral and cell-mediated responses against *S*. *aureus*; in fact, it is likely that the addition of heat-killed *E*. *coli* may have reoriented the response towards the Th2 pathway, as was seen with the IgG isotypes ratios and cytokine production assays. Responses to *E*. *coli* or *S*. *aureus* IMIs were lately demonstrated to be very contrasting [38,39], at least partly because of the high inflammatory response to LPS found in Gram negative bacteria [40], whereas *S*. *aureus* can modulate and subverts host responses by suppressing pro-inflammatory pathways [41]. This immunomodulation is quickly followed by the invasion and persistence in host cells, allowing the pathogen to maintain infections for extended periods.

Other ways to improve cell-mediated responses have been experimented in cows. It was shown that antigen-specific Th1 and Th17 inflammatory responses are possible following intramammary immunization of cows with a sensitizing protein [42]. Because of its effect on neutrophil activity, an improved Th17 response could represent an interesting way of enhancing phagocytic activity in the mammary gland, since neutrophils represent the dominant defense in the udder against mastitis-causing pathogens [31]. A recent bovine mastitis vaccination study using intranasal inoculation of cows with purified IsdA and ClfA-cholera toxin A2/B chimeras was attempted in order to stimulate mucosal immunity of the mammary gland [43]. The vaccine induced IL-4 expression but not IFN-γ or IL-17 in peripheral blood mononuclear cells of cows 60 days after the trial. The protection efficacy against *S*. *aureus* is however still to be determined.

Some adjuvants have the ability to raise balanced and mixed Th1/Th17 responses [30,44] and current research with mice models show interesting candidates using Toll-like receptors (TLRs) agonists, notably TLR-7 which can recognize single-stranded RNAs [45]. In many ways, a bacterial live vaccine can act as an adjuvant by itself because it can stimulate innate immunity in a broad and powerful manner by providing different ligands to these pattern recognition receptors. Interestingly, studies in human have demonstrated that the mechanism behind the high efficacy of live versus killed vaccines resides in the recognition of bacterial viability through the sensing of bacterial RNA by antigen-presenting cells (APCs) TLR8 [46]. APCs then promote differentiation of follicular T helper cells which are essential actors of B-cells activation, affinity maturation and maintenance of humoral memory [47].

Live vaccines are however a source of concern over their safety, thus finding new ways to engineer powerful and stable attenuations without lowering immunogenicity is of great importance for attenuated vaccine development [48]. For this purpose, we previously established the SCV vaccine double mutant strain based on the interruption of gene *vraG*, of importance for oxidative stress and cationic peptide resistance [49–51] and for virulence in bovine IMIs [25], together with the deletion of gene *hemB* which confers a genetically stable SCV phenotype; combined mutations were found to have a large effect on virulence and survival of the parental strain [24]. Moreover, by using a live-attenuated SCV as vaccine, as opposed to a *S*. *aureus* strain of the normal phenotype, the experimental vaccine from the present study should have the additional advantages of stimulating host cells like if it was infected by both an extra and intracellular pathogen. It is well known that SCVs have their own specific gene expression profile, with highly expressed virulence factors involved in colonization such as adhesins, biofilm production and host cell invasion [14,52,53], whereas the normal phenotype is mostly extracellular and is equipped for dissemination [54]. Since this phenotype switching is a dynamic process that naturally occurs during infections [52,55], increasing the host immune response against the SCV phenotype means that this response could be better suited to recognize the specific *in vivo* antigens exhibited by SCVs during persistence as well as stimulating more efficiently the cellular response. Yet, it was previously demonstrated that the live SCV vaccine shared enough features with the normal phenotype to elicit by immunization of mice a high-titer antibody response able to recognize non-SCV clinical strains from different *S*. *aureus spa* types [24]. Besides, *in vivo* experiments in mice models do not always translate in cattle, just in the same way as successes of preclinical studies do not always reproduce in clinical trials. Murine models are useful to compare and select multiple vaccine formulations before evaluation of a selected subset in cows. Ovine models [56] might also provide an additional predictive value for efficacy in cows.

On a final note, caution should be used when inducing a robust Th17 response, notably because of its implication in autoimmune diseases, as was previously discussed [3]. However, the development of a vaccine to prevent *S*. *aureus* infections in humans as opposed to one for bovine mastitis may overall require different strategies, since *S*. *aureus* IMIs rarely become life-threatening systemic infections in cows. The subclinical and recurrent aspects of bovine mastitis represent important challenges for milk producers and a vaccine that reduces dissemination during milking, the occurrence of new infections or the duration of IMIs would economically be advantageous, in contrast to a vaccine for humans, which would be required to significantly reduce disease severity or to prevent systemic infections.

In summary, the live-attenuated *S*. *aureus* SCV vaccine was found to surpass the heat-killed formulation in its ability to raise specific and balanced humoral and cell-mediated responses against *S*. *aureus*. Besides, addition of the inactivated *E*. *coli* J5 bacterin to the vaccine offered no benefit regarding the immune response against *S*. *aureus* as it lowered IgG titers, shifted IgG isotypes towards Th2 response and greatly reduced the production of IFN-γ and IL-17a cytokines from stimulated mouse splenocytes. A strong and balanced immune response is likely the key for handling persistent and recurrent *S*. *aureus* IMIs. A vaccine based on a live-attenuated SCV could possibly significantly improve protection efficacy against *S*. *aureus*. A vaccine that could successfully lead to the elimination of *S*. *aureus* in the early stages of colonization of the udder should bring down transmission rates and eliminate reservoirs of new infections. This would be the key for maintaining and improving long-term competitiveness of milk producers.
